# The impact of cycle proficiency training on cycle-related behaviours and accidents in adolescence: findings from ALSPAC, a UK longitudinal cohort

**DOI:** 10.1186/s12889-016-3138-2

**Published:** 2016-06-09

**Authors:** Alison Teyhan, Rosie Cornish, Andy Boyd, Mary Sissons Joshi, John Macleod

**Affiliations:** School of Social and Community Medicine, University of Bristol, Oakfield House, Oakfield Grove, Bristol, BS8 2BN UK; Department of Psychology, Social Work & Public Health, Oxford Brookes University, Oxford, UK

**Keywords:** Cycle proficiency training, Bikeability, Evaluation, Adolescence, Cycle helmets, High-visibility clothing, Injury prevention, Hospital attendance, ALSPAC

## Abstract

**Background:**

Cycle accidents are a common cause of physical injury in children and adolescents. Education is one strategy to reduce cycle-related injuries. In the UK, some children undertake National Cycle Proficiency Scheme [NCPS] training (now known as Bikeability) in their final years of primary school. It aims to promote cycling and safe cycling behaviours but there has been little scientific evaluation of its effectiveness.

**Methods:**

The sample (*n* = 5415) were participants in the Avon Longitudinal Study of Parents and Children who reported whether or not they had received NCPS training. Outcomes were self-reported at 14 and 16 years: cycling to school, ownership of cycle helmet, use of cycle helmet and high-visibility clothing on last cycle, and involvement in a cycle accident. An additional outcome, hospital admittance due to a cycle accident from 11 to 16 years, was also included for a subsample (*n* = 2222) who have been linked to Hospital Episode Statistics (HES) data.

**Results:**

Approximately 40 % of the sample had received NCPS training. Trained children were more likely to cycle to school and to own a cycle helmet at both 14 and 16 years, to have worn a helmet on their last cycle at age 14, and to have worn high-visibility clothing at age 16, than those who had not attended a course. NCPS training was not associated with self-reported involvement in a cycle accident, and only six of those with HES data had been admitted to hospital due to a cycle accident. Irrespective of training, results indicate very low use of high-visibility clothing, very few girls cycling as part of their school commute, and less than half of helmet owners wearing one on their last cycle.

**Conclusions:**

Our results suggest cycle training courses for children can have benefits that persist into adolescence. However, the low use of cycle helmets, very low use of high-visibility clothing, and low levels of cycling to school for girls, indicate the further potential for interventions to encourage cycling, and safe cycling behaviours, in young people.

**Electronic supplementary material:**

The online version of this article (doi:10.1186/s12889-016-3138-2) contains supplementary material, which is available to authorized users.

## Background

Cycling is beneficial for an individual’s health, and has wider benefits for society and the environment when it replaces motorised transport [1–3]. Cycling is particularly popular with children, both for recreation and as a means of transportation [4]. However, cycle-related injuries are one of the most common causes of physical injury in children. In the UK, almost 2000 child cyclists were injured in road traffic accidents in 2013, and 6 were killed [5]. These figures only include incidents reported to the police, and do not include those which occur away from the road; whilst the number of deaths is accurate, the true number of injuries is thought to be two to three times higher [5]. Children aged 10–15 years are at greater risk of having a cycling accident than any age group other than adults aged over 60 years [5].

Education is one strategy to reduce cycle-related injuries, and many countries have practical cycle training courses for children. In the UK, the National Cycle Proficiency Scheme (NCPS) was first introduced in 1947, and 40 % of children participate by their twelfth birthday [6, 7]. The NCPS was rebranded ‘Bikeability’ in 2007 [8]. As NCPS courses were the responsibility of Local Authorities, content and delivery varied by area, but all adhered to the same guidelines and had the over-arching aim to ‘enable people to cycle safely and to promote cycling by improving skills, knowledge, attitudes, behaviour and hazard awareness’ [9]. A typical course took place during the final years of primary school, consisted of 4–8 sessions each lasting between 1 and 1.5 h (which usually took place in the school playground or on road), and ended with a Cycle Proficiency Test to assess if the children had mastered the skills taught [6].

Despite the significant resources invested in child cycle training in the UK and elsewhere, there has been little scientific evaluation of their outcomes [10, 11]. As children’s knowledge of road safety is often not translated into behaviour [10], it has been argued that it is better to assess safety by measuring behaviour, or outcomes that may be influenced by safety behaviour, than by measuring knowledge [6]. Few UK studies have evaluated whether the NCPS achieved its wider aims in the longer term, such as reducing injuries, promoting bike use, or improving safety behaviours [7]. In one study of almost 2000 children, the NCPS was associated with improved cycling skills and knowledge, with the effect lasting at least two years after training [6]. However, in a study of 336 15 year olds, there was no evidence that NCPS training produced safer attitudes to cycling or reduced accidents [12]. A recent Bikeability evaluation assessed children before, immediately after, and two months after their training and compared them to untrained children: children who had received Bikeability training scored higher on a ‘hazard perception and appropriate response’ quiz than children who had not received training, both immediately and two months after training [13]. However, the practical skills of the trained children reduced over the two month follow-up period, suggesting the children struggled to put their new knowledge into practice [13].

In this current study, we use a longitudinal, population-based cohort to examine whether NCPS training is associated with cycling, cycling safety behaviours, or accidents in adolescence. Specifically, we wanted to determine if NCPS training is associated with (1) cycling to school, (2) use of cycle helmets or high-visibility clothing, and (3) cycle accidents.

Note that we use the word ‘accident’ in this paper when referring to our own data and results because this was the wording used both in the questionnaires completed by the participants, and in the coding of the hospital admissions data. We also use the term when referring to the results of previous studies, and routine statistics, which have used it. However, we acknowledge that the word is problematic as it often implies an unpredictable event, due to bad luck or chance. This is not true of many ‘accidents’, which are often both predictable and preventable [14].

## Methods

### Sample

The sample were participants in the Avon Longitudinal Study of Parents and Children (ALSPAC), a birth cohort study which recruited 14,541 pregnant women with expected delivery dates between April 1, 1991 and December 31, 1992 and who lived in a defined area in and around the city of Bristol, UK. There were 13,988 children alive at one year of age. The children have been studied throughout their lives using maternal and child questionnaires, and clinic visits. A cohort profile gives further details [15] and a searchable data dictionary is available [16]. The study sample in this paper comprises the 5415 singleton children (2462 boys and 2953 girls) who reported whether they had ever received cycle proficiency training and whose Year 6 school was known. Ethical approval for ALSPAC was obtained from the ALSPAC Ethics and Law Committee and the Local Research Ethics Committees (LREC). Full LREC details are available online (http://www.bristol.ac.uk/alspac/researchers/research-ethics/). This study was approved by the ALSPAC Executive Committee. ALSPAC participants who complete questionnaires consent to the use of their data by approved researchers. Participants have the right to withdraw their consent for elements of the study, or from the study entirely, at any time.

### Measures

#### Exposure

The adolescents, at a mean age of 13.9 years, reported whether they had ever been on a Cycle Proficiency Training Course (no/don’t know, yes).

#### Outcomes

##### Self-reported

The adolescents reported their cycle-related behaviours and accidents at 13.9 years and 16.7 years (referred to hereafter as 14 years and 16 years for simplicity): own a cycle helmet (no, yes); wore a cycle helmet last time you rode a bike (no/can’t remember, yes); wore reflective/fluorescent clothing last time you rode a bike (no/can’t remember, yes); ever currently cycle as part of school commute (no, yes); been in a road accident as a cyclist in past year (no, yes).

##### Hospital attendance

A sub-sample of the ALSPAC cohort has been linked to the Hospital Episode Statistics (HES) dataset compiled by the NHS Health and Social Care Information Centre (© 2012, re-used with the permission of The Health and Social Care Information Centre, all rights reserved.) This sub-sample is restricted to ALSPAC participants who, via a postal consent campaign conducted from 2011 to 2013, explicitly consented to the extraction and use of their NHS health records by ALSPAC (details on this sub-sample, and the consent campaign have been published previously [17]). Of the 5414 participants in our sample, 2222 (41 %) have been linked to the HES dataset. The binary outcome (no, yes) was hospital admittance due to a cycle accident (as defined by ICD-10 codes V10-V19 ‘pedal cyclist injured in transport accident’) from the August of the year the child finished Year 6 until the August of the year they finished Year 11 (approximately age 11–16 years).

#### Other variables

Child-reported variables included sex and age at data-collection, bike ownership (no, yes), when last cycled (in last week, in last month, >1 month ago), and distance last cycled (<1 mile, 1–3 miles, >3–5 miles, >5 miles). Proxy measures of socioeconomic position (SEP) were reported by the mother during pregnancy: highest maternal education (university degree; A level; O level; vocational/none); financial difficulties (quartiles of score with range 0–40, where 0 is no financial difficulties); housing tenure (owned/mortgaged, private rent, council rent, other); and highest parental occupational social class (higher of mother and her partner) based on the job codes of the Office for Population Censuses and Surveys [18] and grouped into 4 categories (I/II [professional/managerial & technical]; IIInm [skilled, non-manual]; IIIm [skilled, manual]; IV/V [semi-skilled/unskilled manual]). Maternal-reported variables also included maternal smoking in pregnancy (no, yes), maternal age at delivery (≤23 years, >23–28 years, >28–33 years, >33 years) and parity (0, 1+). When the child was aged 10 years, the mother reported whether the child lived with their father (biological father, non-biological father, no) and whether they lived with older siblings (no, 1, 2+). A pseudo-ID for school attended in Year 6 was identified through linkage to the National Pupil Database.

### Missing data

Multiple imputation using chained equations was used to replace missing data (summarised in Additional file 1: Table A) with predictions based on information observed in the sample. All of the variables included in the analysis models, including outcome variables at both 14 and 16 years, plus other variables predictive of missingness (e.g., measures of child behaviour), were included in the imputation model. One hundred imputed datasets were created and analyzed using ‘mi estimate’ commands in Stata 13 (StataCorp, College Station, Texas). Complete case analyses (*n* = 2094: 877 boys, 1217 girls) was also conducted; results were comparable to those obtained from the imputed data and are available from the authors on request.

### Statistical analyses

Analyses of cycle helmet ownership, use of fluorescent/reflective clothing, and cycling to school, were restricted to children who owned a bike. Analysis of cycle helmet use was restricted to those who owned a bike and helmet. First, descriptive analysis was conducted to compare bike ownership, cycle and safety behaviours by sex and age, and to compare the characteristics of the children who attended and did not attend cycle proficiency training. The association between cycle proficiency training and each of the outcomes was then analysed using either multilevel logistic regression (individual at level 1, school at level 2) or standard logistic regression, dependent on whether there was statistical clustering at the school level. For each outcome, two models were fitted. Model 1 adjusted for age and sex. Model 2 also included maternal age at delivery, parity, and the SEP (parental social class; maternal education; financial difficulties; housing tenure; maternal smoking) and family (lives with biological dad; lives with older siblings) measures. For use of a cycle helmet and use of fluorescent/reflective clothing, a third model also adjusted for characteristics of the last cycle (length and when it took place). Interaction terms were fitted to test whether the relationship between cycle proficiency training and the outcomes differed by child sex: these were not significant and so models were adjusted for sex but not stratified.

## Results

Bike ownership was more common in boys than girls, but declined with age for both genders (Table 1). Cycling behaviour also changed with age: cycling in the past week was more common at 14 than 16 years, but the length of the last cycle increased with age. Girls were less likely to have cycled in the past week than boys, and were more likely to have cycled a short distance on their last ride. Less than 2 % of girls reported cycling as part of their school commute, compared to over 10 % of boys.

**Table 1:** Bike ownership, cycle and safety behaviours, and accidents by gender and age

		Boys (*n* = 2462)		Girls (*n* = 2953)	
		14 years	16 years	14 years	16 years
Has own bike	Yes (%)	95.0 (94.1–95.9)	85.8 (84.0–87.6)	87.8 (86.5–88.9)	71.2 (69.2–73.1)
When last cycled	In last week (%)	54.4 (52.4–56.4)	36.5 (34.1–39.0)	21.8 (20.3–23.2)	8.1 (7.0–9.3)
	In last month (%)	21.3 (19.7–22.9)	15.9 (14.1–17.7)	22.5 (21.0–24.0)	9.7 (8.5–10.9)
	>1 mth ago (%)	24.3 (22.6–26.0)	47.6 (45.1–50.0)	55.8 (54.0–57.5)	82.2 (80.6–83.7)
Distance last cycled	<1 mile (%)	29.1 (27.3–30.9)	20.9 (18.7–23.0)	36.2 (34.5–38.0)	26.6 (24.7–28.4)
	1–3 miles (%)	49.1 (47.1–51.1)	45.0 (42.5–47.4)	41.8 (40.0–43.6)	44.6 (42.4–46.8)
	3–5miles (%)	10.4 (9.2–11.6)	15.1 (13.3–17.0)	11.4 (10.2–12.5)	14.1 (12.6–15.5)
	>5 miles (%)	11.3 (10.1–12.6)	19.0 (17.0–21.0)	10.6 (9.5–11.8)	14.8 (13.2–16.3)
Cycles as part/all of school commute	Yes (%)	12.9 (11.5–14.2)	14.5 (12.7–16.3)	1.9 (1.4–2.3)	1.6 (1.1–2.1)
		*n* = 2337	*n* = 2079	*n* = 2585	*n* = 2072
Wore fluorescent/reflective clothing on last cycle^a^	Yes (%)	4.5 (3.6–5.3)	7.2 (5.7–8.6)	3.1 (2.4–3.8)	4.2 (3.2–5.3)
Owns cycle helmet^a^	Yes (%)	65.1 (63.2–67.0)	56.6 (53.9–59.3)	62.5 (60.7–64.4)	53.9 (51.6–56.3)
		*n* = 1520	*n* = 1150	*n* = 1614	*n* = 1105
Wore cycle helmet on last cycle^b^	Yes (%)	41.6 (39.2–44.1)	41.0 (37.6–44.3)	40.6 (38.2–43.0)	44.3 (41.0–47.6)
Had a bike accident in previous 12 months at 14 or 16 years	Yes (%)	4.4 (3.0–5.9)		2.1 (1.4–2.8)	

^a^Restricted to those who own a bike

^b^Restricted to those who own a bike and a cycle helmet

The use of fluorescent/reflective clothing was low, but increased with age, particularly for boys (Table 1). Over half of children who owned a bike also owned a helmet, but only around 40 % of those with a bike and a helmet had worn the helmet on their last cycle ride. Cycle helmet ownership was higher at 14 than 16 years, but amongst those who owned a helmet the proportion of children wearing one on their last ride was similar at both ages. Self-reported cycle accidents were rare, but more common in boys than girls. Of the 2222 participants linked to HES data, only six had been admitted to hospital due to injuries obtained while cycling.

Although the majority of children owned a bike, less than half reported that they had received cycle proficiency training: 42.0 % (95 % CI 40.0–43.9) of boys and 38.0 % (95 % CI 36.2–39.7) of girls. Compared to those who had not had cycle proficiency training, those who had were more likely to be male, and their families to be of higher SEP (Table 2). Children who had attended training were more likely to own a bike at both 14 and 16 years (Additional file 1: Table B).

**Table 2:** Sample description by cycle proficiency training status

		Had cycle proficiency training		
		Yes (*n* = 2156)	No (*n* = 3259)	*p*-value
Sex	Female (%)	52.0 (49.9–54.2)	56.2 (54.5–57.9)	0.003
Maternal education	Degree (%)	20.1 (18.4–21.8)	13.0 (11.8–14.2)	<0.0001
	A level (%)	28.8 (26.9–30.8)	24.1 (22.6–25.6)	
	O level (%)	33.6 (31.5–35.6)	37.3 (35.6–39.0)	
	None/vocational (%)	17.5 (15.9–19.1)	25.7 (24.1–27.2)	
Financial difficulties	Q1 (none) (%)	44.7 (42.5–46.8)	38.5 (36.8–40.3)	<0.0001
	Q4 (%)	12.0 (10.6–13.4)	18.0 (16.6–19.3)	
Highest parental occupational social class	I & II (%)	65.7 (63.6–67.7)	55.6 (53.8–57.3)	<0.0001
	IIInm (%)	23.0 (21.2–24.9)	27.5 (25.9–29.1)	
	IIIm (%)	8.4 (7.2–9.6)	11.7 (10.5–12.9)	
	IV & V (%)	2.9 (2.2–3.7)	5.2 (4.4–6.1)	
Housing tenure	Owned/mortgaged (%)	85.5 (84.0–87.0)	81.3 (80.0–82.7)	0.0001
	Rent - private (%)	4.8 (3.9–5.7)	5.4 (4.6–6.2)	
	Rent - council (%)	6.9 (5.8–8.0)	10.5 (9.4–11.5)	
	Other (%)	2.9 (2.1–3.6)	2.9 (2.3–3.4)	
Maternal smoking	Yes (%)	15.2 (13.7–16.7)	20.3 (18.9–21.7)	<0.0001
Parity	1+ (%)	50.5 (48.4–52.7)	54.2 (52.4–55.9)	0.009
Maternal age at delivery	<=23 yrs (%)	7.7 (6.6–8.9)	12.5 (36.3–39.7)	<0.0001
	>23 to < =28 yrs (%)	35.3 (33.3–37.4)	38.0 (36.3–39.7)	
	>28 to < =33 yrs (%)	39.2 (37.1–41.3)	34.9 (33.3–36.6)	
	>33 yrs (%)	17.7 (16.1–19.3)	14.6 (13.4–15.8)	
Older siblings at home	None (%)	49.2 (47.0–51.3)	45.9 (44.1–47.6)	0.018
	1 (%)	36.5 (34.3–38.6)	37.1 (35.4–38.8)	
	2+ (%)	14.4 (12.8–15.9)	17.0 (15.6–18.4)	
Resident dad	Biological (%)	84.4 (82.8–86.1)	77.1 (75.5–78.7)	<0.0001
	Non-biological (%)	6.4 (5.3–7.6)	9.4 (8.3–10.5)	
	No (%)	9.1 (7.8–10.4)	13.5 (12.2–14.8)	

Cycle proficiency training was associated with cycle helmet ownership at both age 14 (75 % of those trained versus 56 % of those not trained) and 16 years (67 % versus 45 %) (Table 3). Amongst those who owned a helmet, children who had attended cycle proficiency training were more likely to have worn the helmet on their last cycle at 14 years (45 % versus 37 %), but not at 16 years (44 % versus 42 %). Those who had attended cycle proficiency training were also more likely to have worn reflective/fluorescent clothing on their last cycle at 16 years (7 % versus 5 %). Adjustment for characteristics of the last ride (length of cycle and how recently it took place) had little impact on the associations observed between cycle proficiency training status and reflective/fluorescent clothing or helmet use. However, those who had cycled further on their last cycle, and those whose last cycle was longer ago, were more likely to have worn a helmet at both 14 and 16 years (Additional file 1: Table C). At 14 years, those who had cycled further were more likely to have worn high-visibility clothing, but those whose last cycle was longer ago were less likely to have worn it. Cycle proficiency training was positively associated with cycling to school at both 14 years (9 % versus 7 %) and 16 years (11 % versus 8 %). The proportion reporting that they had had a cycle accident did not differ by cycle proficiency training status (Table 3). The small numbers admitted to hospital as a result of a cycle accident prevented further analysis of this outcome.

**Table 3:** Association between cycle proficiency training and cycle safety behaviours and accidents

					OR (95 % CI); p-value		
Outcome	Age	Attended cycle proficiency training?	*n*	% (95 % CI) reporting outcome	Model 1^a^ Sex and age	Model 2^b^ Model 1 + SEP, family variables	Model 3^c^ Model 2 + cycle characteristics
Own helmet^d^	14	No	2881	55.9 (54.1–57.7)	Ref	Ref	
		Yes	2041	74.8 (72.9–76.7)	2.38 (2.05–2.76); *p* < 0.0005	2.06 (1.78–2.39); *p* < 0.0005	/
	16	No	2347	46.6 (44.2–49.0)	Ref	Ref	
		Yes	1779	66.8 (64.2–69.4)	2.34 (1.96–2.78); *p* < 0.0005	2.03 (1.72–2.41); *p* < 0.0005	/
Wore helmet^e^	14	No	1609	37.4 (35.1–39.8)	Ref	Ref	Ref
		Yes	1525	45.0 (42.5–47.5)	1.33 (1.13–1.57); *p* = 0.001	1.26 (1.07–1.49); *p* = 0.005	1.25 (1.06–1.48); *p* = 0.010
	16	No	1070	41.6 (38.1–45.0)	Ref	Ref	Ref
		Yes	1176	43.6 (40.4–46.8)	1.09 (0.90–1.32); *p* = 0.394	1.04 (0.85–1.27); *p* = 0.716	1.03 (0.84–1.27); *p* = 0.751
Wore fluorescent/reflective clothing^d^	14	No	2881	3.3 (2.6–4.0)	Ref	Ref	Ref
		Yes	2041	4.4 (3.5–5.3)	1.33 (0.98–1.79); *p* = 0.064	1.34 (0.98–1.81); *p* = 0.063	1.28 (0.94–1.74); *p* = 0.118
	16	No	2347	4.6 (3.4–5.8)	Ref	Ref	Ref
		Yes	1779	7.2 (5.8–8.6)	1.60 (1.15–2.23); *p* = 0.005	1.70 (1.22–2.39); *p* = 0.002	1.68 (1.20–2.35); *p* = 0.003
Cycled to school^d^	14	No	2881	6.7 (5.8–7.6)	Ref	Ref	
		Yes	2041	8.7 (7.5–9.9)	1.40 (1.08–1.80); *p* = 0.010	1.56 (1.20–2.02); *p* = 0.001	/
	16	No	2347	8.2 (6.8–9.7)			
		Yes	1779	10.9 (9.2–12.6)	1.36 (1.03–1.79); *p* = 0.033	1.48 (1.11–1.97); *p* = 0.008	/
Any cycle accident in past year at 14 or 16 years		No	3259	3.1 (2.1–4.2)	Ref	Ref	/
		Yes	2156	3.2 (2.0–4.3)	0.99 (0.59–1.68); *p* = 0.977	1.04 (0.61–1.78); *p* = 0.883	/

^a^Model 1: age, sex

^b^Model 2: Model 1 + occupational social class, maternal education, financial difficulties, housing tenure, maternal age at delivery, maternal smoking, parity, older siblings, resident father

^c^Model 3: Model 2 + distance last cycled, when last cycled (for ‘wore helmet’ and ‘wore reflective/fluorescent clothing’ outcomes only)

^d^Restricted to those who own their own bike

^e^Restricted to those who own a bike and a cycle helmet

## Discussion

Cycle proficiency training was associated with some cycle-related safety behaviours in adolescence, and with cycling to school. However, it was not associated with self-reported involvement in a road traffic accident as a cyclist. A very small number of children had been admitted to hospital due to a cycle-related injury, preventing analysis of this outcome by cycle proficiency training status. Bike ownership was very common in our sample of adolescents yet less than half had received NCPS training, a proportion similar to that observed nationally [12]. NCPS training was not available in all schools, and this remains the situation today. It is estimated that in 2013/2014 only around half of primary schools offered Bikeability training, and these schools are not evenly distributed across the country [19].

### Ownership and use of safety equipment

Children who attended an NCPS course were more likely to own a cycle helmet in adolescence, and to have used a helmet on their last cycle at age 14, than those who had not attended a course. This is consistent with a previous study of adolescents in their first year of secondary school, which found those who had had NCPS training were more likely to own and use a cycle helmet [6]. Head injuries are the greatest health risk posed to cyclists [5], and there is substantial evidence that helmets reduce the risk of head, brain and facial injuries, and death, in cyclists involved in a crash [20, 21]. In the UK, there is no law compelling cyclists of any age to wear a helmet, although the Highway Code states that cyclists should wear one [22]. The relatively high level of helmet ownership in those who attended training in our sample may reflect mandatory helmet use during training in some schools, but we have no information on this.

At 14 years those who had attended training were more likely to have worn a helmet, suggesting a beneficial impact of training on helmet use that persists for at least a couple of years. In contrast, a previous study that used the ALSPAC sample to evaluate a wide-ranging safety training programme, where cycle safety is one of many topics covered, found no association between attendance and cycle helmet ownership or use [23]. This suggests the NCPS, with its practical nature and sole focus on cycle safety, is a more effective way of promoting some cycle safety behaviours. The increased helmet usage associated with NCPS training could be due to higher levels of knowledge of the safety benefits in trained children. However a study based in Oxford, a city with a high rate of cycling [24], found that almost all teenagers knew wearing a helmet reduced the risk of head injury [25]. Health behaviour models have long indicated that knowledge, while necessary, may not be sufficient to result in behaviour change as it competes with other barriers, such as social and behavioural norms [26, 27]. In the Oxford study for example, over 70 % thought helmets ‘looked ridiculous’, and many said their friends ‘discouraged them from wearing one’ [25]. The positive impact of the NCPS on helmet use could perhaps result from helping establish such behaviour as normal in a peer group. By 16 years, helmet use in those who had not attended training had reached similar levels to those who had. However, irrespective of training attendance, less than half of those who owned a helmet wore it on their last cycle ride. In concordance with this, a study which assessed children’s practical cycling skills found only 27 % of those who owned a helmet brought it with them for the test [6]. In our sample, there was a positive association between distance last cycled and helmet use on that cycle; this suggests people may wrongly assume short journeys are safer. Furthermore, those whose last cycle was a longer time ago were more likely to have worn a helmet, perhaps indicating that those who cycle regularly perceive cycling as safer than those who cycle less regularly.

Use of high-visibility clothing was very low overall, although higher at 16 years than 14 years. This may reflect older adolescents being more likely to cycle for transportation, to cover longer distances, and to be on the roads in low light or dark conditions. Those who had attended cycle proficiency training were more likely to have worn reflective or fluorescent clothing, particularly at 16 years. At 14 years, those whose last cycle was a longer time ago were less likely to have worn high-visibility clothing; less frequent cyclists may be less likely to own such equipment. In the UK, there is no legal requirement for cyclists to wear high-visibility clothing but the Highway Code states cyclists should wear light-coloured or fluorescent clothing in daylight and poor light, and reflective clothing and/or accessories (e.g., ankle bands) in the dark [22]. High-visibility clothing aims to increase cyclists’ visibility and conspicuity on the road, and hence reduce collisions, which are often due to car drivers failing to see a cyclist in time [28]. The benefits of reflective versus fluorescent clothing depend on the time of day; fluorescent clothing is a useful daytime visibility aid but is of little use in darkness, conversely reflective clothing can greatly improve visibility at night [29, 30]. Although many cyclists are aware of the visibility benefits of such clothing, studies have found low usage in both adults and children [29, 31, 32]. This may be partly due to cyclists, like pedestrians, not fully appreciating their ‘invisibility’ relative to cars [28]. Conversely, there is a danger that those who do wear such clothing over-estimate the effect it has on their visibility, particularly those wearing fluorescent clothing at night [29, 30].

In focusing on low usage of helmets and reflective/fluorescent clothing in our sample, it is worth noting that in countries with high rates of cycling (e.g., the Netherlands, Denmark, and Germany), the use of such protective equipment is low [33]. The safety focus in these countries is on the physical separation of bikes and cars on busy roads and intersections, together with traffic calming measures in residential areas [33]. Additionally, there is evidence that the use of such safety equipment does not improve driver behaviour. One study found no evidence that use of high-visibility clothing changed drivers overtaking behaviour [34], and the impact of high-visibility clothing on cyclist safety is unknown [35]. Furthermore, drivers have been found to leave less space when overtaking cyclists wearing helmets [36]. It is therefore important that young cyclists are not given a false sense of security from the use of such safety equipment.

### Cycle accidents

It has been argued that accidents are too rare to use as an outcome to assess the efficacy of cycle training courses [12]. In our sample, very few of the adolescents reported being involved in a road traffic accident as a cyclist. However, figures from Public Health England (2012/13) indicate that admissions to hospital via A&E for non-vehicular cycle accidents outnumbered cycle accidents involving another vehicle by approximately 7 to 1 for 10–13 year olds. This suggests that the ALSPAC participants may have been involved in many more accidents than those reported, if the question was interpreted as referring only to accidents involving a vehicle. In support of this, in a previous evaluation of the NCPS, over half of the accidents did not involve another vehicle (i.e., the child ‘just fell off’) [6]. However, the total number of accidents was also small in this study and precluded any detailed analyses. Another NCPS evaluation reported much higher numbers of accidents: when 15 year olds were asked if they had ever been in a cycle accident, almost 18 % reported that they had been in one which required attendance at hospital, 45 % had had a minor accident, and 60 % had had a near miss while cycling [12]. These higher percentages are likely due to the time frame being ‘ever’ as opposed to the previous 12 months as in our study, and the definition of accidents not being limited to road traffic accidents. Nevertheless, consistent with our study, boys were more likely to report an accident than girls, and there was no association between NCPS training and involvement in an accident. In considering whether training can have a positive impact on prevention of accidents, it is important to consider the potential for unintended negative consequences; an Australian study found that trained children, particularly boys, were more likely to be injured when cycling than untrained children, perhaps due to increased risk taking and reduced supervision [37].

### Cycling to school

Active transport to school is an important source of physical activity in young people, and cycling to school has been shown to improve the cardiovascular health of children and adolescents [38–40]. Cycling also has wider benefits, including being environmentally friendly, economical, and giving young people independence [33]. A key aim of the NCPS was to ‘promote and encourage’ cycling [9], and the UK government have stated that they want to ‘encourage more people to cycle more safely and more often’ [41]. We found that children who had attended training were more likely to report cycling as part of their school commute at both 14 and 16 years. Evaluations of the impact of Bikeability on cycling frequency have had mixed results. One study found that in local authorities in England where there is a longer history of delivering cycle training to primary school children, a higher proportion of children cycle to secondary school [42], and a further study found children and their parents both reported that the children cycled more often after they received training [4]. However, two studies that compared cycling rates in trained and untrained children did not have such positive results. One used two datasets to assess children’s cycling behaviour, and found a positive association with Bikeability training in one dataset but not the other [19]. The other study found that two months after the course finished, the trained children did not report cycling more frequently than the untrained children, despite reporting that training had made them feel more confident about cycling on the road [13].

Very few of the girls in our study cycled to school, and they reported that their last cycle was shorter and a longer time ago than the boys. This is consistent with previous studies [12, 43–46], and is also the gender pattern observed in adults in countries with a low cycling prevalence [47–49]. Barriers and incentives to cycle likely differ by gender and age. For example, boys who cycle to school may be attracted to cycling not just as a mode of transport but as a physical activity in its own right [46]. In contrast, perceived safety issues may be of more concern to girls, as indicated by a study in Melbourne suggesting that female cyclists prefer to use routes with maximum separation from motorized traffic [50].

In the 2014 National Travel Survey in England, only 1 % of all journeys were made by bike [49] (as way of comparison, over a quarter of all journeys are by bike in the Netherlands [51]). There is clearly a long way to go in establishing cycling as a viable alternative to other forms of transport in the UK for both children and adults. It is worth highlighting that ALSPAC is based in and around Bristol, a city with relatively high rates of cycling by UK standards [24]. Bristol was designated a ‘cycling city’ by the Government in 2008, the first UK city to be given this status, and consequently awarded £22 million to invest in cycling [52]. Since 2009 the city council have measured cycling behaviour in its annual ‘Quality of Life in Bristol’ survey, and in 2014 noted an increase for the first time: 24 % of respondents cycled at least once a week (versus 19 % in 2009), and 16 % cycled to work (versus 9 % in 2009) [53]. However, rates of cycling vary substantially between different parts of the city: over 40 % of those in some areas cycle at least once a week, compared to less than 9 % in other areas [53]. The contextual effect of living in an area where there is appropriate infrastructure to enable safe cycling, and where travelling by bike is viewed positively and as a social norm, could ultimately have as much influence on whether or not adolescents cycle, and their cycling behaviours, as do cycle training courses. However, it can be argued that a high rate of cycling, as in the Bristol area, renders cycle training especially important as there is some evidence, in the comparable area of car driving, that young people may acquire poor safety habits from observing their parents’ everyday behaviours [54].

### Limitations

Our study has limitations and the results should be interpreted in light of these. None of the ALSPAC measures were designed *a priori* to evaluate the NCPS. The children were not asked for any details of the training they had undertaken, therefore we were unable to consider whether outcomes differed between those who were trained in a playground compared to those who trained on roads, for example. We have no measure on how the child performed during their training or whether they passed their Cycle Proficiency Test (although pass rates are generally high [12]). The measures that relate to use of a cycle helmet and fluorescent/reflective clothing refer only to the last time cycled and may not reflect typical use. We have no information on the purpose of that last cycle ride, or the time of day. Participants were asked about accidents in the 12 months prior to data collection; as the period between the two outcome time points was over two years, we will have missed some accidents. We had no information on near misses while cycling: near misses are markedly more frequent than collisions and can have a substantial impact on an individual’s cycling experience [55, 56]. Our hospital admissions outcome probably only captures individuals who sustained the most serious injuries; more minor cycle injuries would have been treated in A&E but a lack of detail in admission codes prevents us from being able to determine which visits to A&E were the consequence of cycle-related injuries. We cannot rule out reverse causation as an explanation for the associations observed as we have no measure of bike use or of cycle safety behaviours in earlier childhood. This was also a limitation of a recent Bikeability evaluation, and signals that more data is needed on the differences between children who do and do not receive cycle training [19]. Finally, our sample undertook their training in approximately 2002–2004; the NCPS has since undergone some changes and been rebranded to Bikeability (although its core aims remain the same) [8].

## Conclusions

In the UK, Bikeability training is a key strategy in ensuring the cycle safety of young people [8]. The training is rated highly by both children and their parents, and the vast majority of parents believe it is very important [4]. Our results suggest cycle training courses for children can have a lasting positive effect on cycling and some related safety behaviours, and that these benefits can persist into adolescence. Nevertheless, our results also indicate low use of cycle helmets and very low use of high-visibility clothing in adolescence. Additionally, very few girls cycled as part of their school commute. There is therefore the potential for interventions to have an even greater impact on encouraging cycling, and safe cycling behaviours, in young people. For interventions to be more successful they will need to address not only rates of cycling and the teaching of safety skills and knowledge, but also confront such biases as ‘unrealistic optimism’ and the assumption of above-average skill, which may mean that for some cyclists safety knowledge is learned but not considered relevant to the self [57, 58]. For knowledge to affect behaviour, hazard awareness needs to be augmented by hazard relevance.

## Abbreviations

ALSPAC, Avon Longitudinal Study of Parents and Children; HES, hospital episode statistics; NCPS, National Cycle Proficiency Scheme

## Additional file

Additional file 1: Supplementary Tables (Tables A, B, and C). (DOCX 15 kb)
