# Supplementary material for: The impact of cycle proficiency training on cycle-related behaviours and accidents in adolescence: findings from ALSPAC, a UK longitudinal cohort
Source: BMC Public Health. 2016 Jun 9;16:469. doi: 10.1186/s12889-016-3138-2 (PMC4899925; doi:10.1186/s12889-016-3138-2)
Supplement: Additional file 1: — Supplementary Tables (Tables A, B, and C). (DOCX 15 kb) [file 12889_2016_3138_MOESM1_ESM.docx]

**Additional File 1**

**Table A: summary of missingness**

| **Variable** | **Time point** | **n (%) missing** |
| --- | --- | --- |
| Sex |  | 0 |
| Maternal age at delivery |  | 0 |
| Maternal education | Pregnancy | 171 (3.2) |
| Highest occupational social class | Pregnancy | 411 (7.6) |
| Housing tenure | Pregnancy | 155 (2.9) |
| Financial difficulties | Pregnancy | 302 (5.6) |
| Maternal smoking | Pregnancy | 94 (1.7) |
| Parity | Pregnancy | 165 (3.1) |
| Number of older siblings in home | 10 years | 878 (16.2) |
| Dad residency status | 10 years | 731 (13.5) |
|  |  |  |
| Bike ownership | 14 years | 33 (0.6) |
|  | 16 years | 1882 (34.8) |
|  |  |  |
| Helmet ownership | 14 years | 50 (0.9) |
|  | 16 years | 1881 (34.7) |
|  |  |  |
| Helmet Use | 14 years | 45 (0.8) |
|  | 16 years | 2052 (37.9) |
|  |  |  |
| Reflective/fluorescent clothing | 14 years | 119 (2.2) |
|  | 16 years | 2049 (37.8) |
|  |  |  |
| Accident as cyclist in past year | 14 years | 1293 (23.9) |
|  | 16 years | 2063 (38.1) |

**Table B: Bike ownership by cycle proficiency training status, gender and age**

|  | **Had cycle proficiency training** | |  |
| --- | --- | --- | --- |
|  | **Yes (n=1034)** | **No (n=1428)** | p-value |
| **Boys** |  |  |  |
| Own bike at 14 years (%) | 96.9 (95.8-98.0) | 93.6 (92.3-94.9) | 0.0003 |
| Own bike at 16 years (%) | 89.1 (87.1-91.5) | 83.3 (80.8-85.8) | 0.0003 |
| **Girls** | Yes (n=1122) | No (n=1831) |  |
| Own bike at 14 years (%) | 92.9 (91.4-94.4) | 84.5 (82.9-86.2) | <0.0001 |
| Own bike at 16 years (%) | 78.9 (76.1-81.7) | 66.5 (63.9-69.0) | <0.0001 |

**Table C: Association between characteristics of last cycle and the use of a helmet and fluorescent/reflective clothing on that cycle.**

|  |  | **14 years** | **16 years** |
| --- | --- | --- | --- |
|  |  | **OR (95% CI)** | **OR (95% CI)** |
| **Wore helmet on last cycle^a^** | | | |
| Distance last cycled | <1 mile | Ref | Ref |
|  | 1-3 miles | 1.48 (1.23-1.78) | 1.25 (0.95-1.65) |
|  | >3-5 miles | 2.01 (1.54-2.63) | 2.27 (1.62-3.20) |
|  | >5 miles | 2.65 (2.03-3.44) | 2.98 (2.14-4.15) |
|  |  |  |  |
| When last cycled | in last week | Ref | Ref |
|  | in last month | 1.52 (1.24-1.87) | 1.20 (0.85-1.70) |
|  | > month ago | 1.46 (1.21-1.77) | 1.48 (1.13-1.92) |
|  |  |  |  |
| **Wore reflective or fluorescent clothing on last cycle^a^** | | | |
| Distance last cycled | <1 mile | Ref | Ref |
|  | 1-3 miles | 2.86 (1.85-4.42) | 1.23 (0.81-1.86) |
|  | >3-5 miles | 1.75 (0.93-3.26) | 1.12 (0.60-2.07) |
|  | >5 miles | 2.80 (1.59-4.92) | 1.19 (0.67-2.14) |
|  |  |  |  |
| When last cycled | in last week | Ref | Ref |
|  | in last month | 0.67 (0.45-1.00) | 0.99 (0.64-1.53) |
|  | > month ago | 0.57 (0.39-0.84) | 0.79 (0.50-1.23) |

^a^Results are from Model 3 (variables in model: sex, age, cycle proficiency training, occupational social class, maternal education, financial difficulties, housing tenure, maternal smoking, parity, older siblings, resident father, distance last cycled, when last cycled).
